# Albert Edward, M. D.

**Published:** 1898-02

**Authors:** 


### Albert Edward, M.D.

The Prince of Wales, at the last meeting of the British Royal College of Physicians having been elected a member of that august body, from now on has the right to commence medical practice in the United Kingdom without interference on the part of the authorities.

